# Understanding the psychodynamic functioning of patients with PTSD and CPTSD: qualitative analysis from the OPD 2 interview

**DOI:** 10.1186/s41155-022-00211-5

**Published:** 2022-04-18

**Authors:** Taís Cristina Favaretto, Luciane Maria Both, Sílvia Pereira da Cruz Benetti, Lúcia Helena Machado Freitas

**Affiliations:** 1 Federal University of Rio Grande do Sul (UFRGS), Av. Paulo Gama 110, Bairro Farroupilha, Porto Alegre, Rio Grande do Sul 90040-060 Brazil; 2 Rua Independência 99a, 401, Centro, Passo Fundo, Rio Grande do Sul 99010041 Brazil; 3 University of Vale do Rio dos Sinos, São Leopoldo, Brazil

**Keywords:** Psychological trauma, Post-traumatic stress disorder, Chronic post-traumatic stress disorder, Violence, Psychoanalytic theory, Qualitative research

## Abstract

The traumatic event produces intolerable excitations to the psychic apparatus that searches to relief them through the production of symptoms. When established, patients with post-traumatic stress disorders (PTSD) and complex post-traumatic stress disorder (CPTSD) may experience flashbacks, somatizations, negative emotions about themselves, and difficulty in social contact. This work seeks to understand how the psychodynamic functioning of women victims of interpersonal and urban violence, diagnosed with these disorders, is organized, identifying traumatic experiences, ways of interpersonal relationships, conflicts and psychic structures and use of defense mechanisms, and for peculiarities that may differentiate these disorders. The qualitative transversal method was used through the content analysis of clinical interviews based on the Operationalized Psychodynamic Diagnosis (OPD-2). The sample of this study consisted of five women with PTSD and five with CPTSD. The following categories were created: reasons for seeking care, symptoms and desire for treatment, traumatic developmental events, and characteristics of the psychic functioning. Early trauma generates psychic organizations with greater disintegration. A new traumatic event destabilizes the psychic organization and intensifies symptoms. Relationships were marked by dependence and isolation. Participants with CPTSD presented tendency to disintegration related to the object relation regulation and the psychic conflict was of Individuation versus Dependence, with more primitive flaws in object representations, existential need for the other and direct discharge of impulses. Participants with PTSD had moderate to low level of object relation integration and the conflict was need to be care of versus self-sufficiency, with self-representations being fragile and with reduced capacity to manage impulses. Thus, it could be observed that OPD-2 is capable of assessing in a broad and deep way patients with traumatic disorders, in addition to identifying essential peculiarities to guide health professionals towards treatment in the search for better quality of life for patients.

**Supplementary Information:**

The online version contains supplementary material available at 10.1186/s41155-022-00211-5.

## Introduction

Post-traumatic stress disorder (PTSD; F43.10) can develop from exposure to or witnessing one or more threatening events or episodes of violence. The presence of intrusive symptoms, overwhelming feelings and avoidance of trauma-related memories (World Health Organization, 2018) are an adaptive response of the psychic apparatus to excessive stress disorders producing considerable subjective suffering (Blanco, 2016).

In 2018, based on clinical observations of individuals who have suffered multiple and/or prolonged traumas of interpersonal nature throughout their lives, a new category for stress-associated disorders was included, namely complex post-traumatic stress disorder (CPTSD; 6B41). CPTSD is characterized by severe and persistent problems with affect regulation, diminished self-beliefs, shame, guilt, or failure related to the traumatic event. Such reactions cause significant damage in different spheres of the subject, including personal, social, and occupational relationships (World Health Organization, 2018).

Such classifications, related to trauma disorders and stressors, defined by the nosological psychiatric diagnosis, seek an objective assessment of symptoms, with concise and clear criteria aimed at adequate treatment (American Psychiatric Association, 2014). On the other hand, psychoanalytic theory seeks to understand human behavior and suffering through the patient’s developmental history, his/her structural constitution of personality and how the dynamics between the mental instances, Id, Ego, and Superego are established. Through psychotherapeutic work, by talking and associating, it seeks to reduce symptoms, change poorly adapted relational patterns, dysfunctional conflicts, and structural limitations, improving quality of life (Campos, 2017).

Their contributions mention psychic trauma as an excessive influx or accumulation of excitations that penetrate the psychic apparatus, exceeding the capacity to face or integrate feelings, in the face of the large amount of stress (violent traumatic event), needing to be relieved (Laplanche & Pontalis, 1991). By breaking through the ego defense mechanisms, this energy puts the developed psychic structure at risk, leading the subject to the position of original helplessness, that is, constantly reliving the newborn’s instinctual anguish in the face of its biological and psychic immaturity (Favero, 2009; Laplanche & Pontalis, 1991; Pereira, 2008). Symptoms appear as a discharge or a substitute for the contents that cause the anguish, unconsciously, the psychic expression of instincts and, consciously, the traumatic event (Costa, 2019).

Pre- and post-traumatic characteristics enable the emergence of potentialities and vulnerabilities in coping with the traumatic event. Protection from trauma arises mainly in the first moments of development with the internalization of representations of good relationships and the constitution of stable self, capable of regulating emotions and behaviors and making use of defense mechanisms in the face of anxiety, with greater flexibility (Eizirik et al., 2015). Traumatic situations such as violence, especially in childhood, can form disorganized psychic structures with failures in the repression of instincts, use of rigid defense mechanisms and low ability to reflect, which makes psychic reorganization difficult in the face of a traumatic event (Bateman & Fonagy, 2016). Furthermore, factors such as intensity, duration, and frequency of trauma (Wilson & Reagan, 2016), social support, physical and mental comorbidities, and socioeconomic issues should also be considered in coping with the traumatic event (Steinert et al., 2015).

Thus, observing the complexity of the human mind and the need to seek an alignment between assessment of patients based on fundamental psychodynamic assumptions and the phenomenological orientation of psychiatric diagnostic glossaries, the Operationalized Psychodynamic Diagnosis instrument (OPD-2) emerges as a means of multiaxial assessment that formulates the way in which patients organize their psychodynamic functioning based on their clinical situation, areas of functioning with symptoms or difficulties, subjective suffering, patterns of interpersonal relationships resulting from intrapsychic conflicts, their resources and competences, deficits in psychological structure and nosological diagnosis. From the integration of axes, it is possible to indicate the focus of the psychotherapeutic treatment focused on the dysfunctional relationship pattern and problems based on conflict and/or structure. For these purposes, the instrument uses as theoretical basis assumptions of the attachment theory and object relation (Task Force, 2016).

Thus, in view of the traumatic disorders that present intense severity of symptoms and extensive damages in the lives of individuals, through OPD-2, this study seeks to understand how the psychodynamic functioning of women victims of interpersonal and urban violence, diagnosed with PTSD and C–PTDS is organized, identifying traumatic experiences, interpersonal relationships, conflicts and psychic structures, and the use of defense mechanisms, in addition to looking for peculiarities that can differentiate these disorders.

## Method

This is a qualitative cross-sectional study whose focus was the content analysis of clinical interviews based on OPD-2. The construction of the study was organized through the Consolidated Criteria for Qualitative Research Reports (Tong et al., 2007). This research is part of a broader study project with subjects who have gone through traumatic events.

### Participants

Participants are ten women from a public health service in a large city in Rio Grande do Sul, Brazil, evaluated in 2019. The place is reference in the evaluation and treatment of trauma victims. Selection was carried out by convenience, among those who were on site at the time of data collection, which took place on alternate days and at different times to achieve greater sample heterogeneity. Five women with PTSD and five with CPTSD were included. Diagnosis was performed through a psychiatric clinical interview present in DSM-5 (American Psychiatric Association, 2014) and ICD-11 diagnostic criteria (World Health Organization, 2018), respectively. The definition of the number of participants was performed by data saturation.

### Instruments

#### Sociodemographic and clinical form

Sociodemographic and clinical form is used to characterize participants, containing data on age, schooling, relationships, and use of psychoactive substances, among others.

#### Operationalized Psychodynamic Diagnosis (OPD-2) clinical interview

Operationalized Psychodynamic Diagnosis (OPD-2) clinical interview is a qualitative analysis of the semi-structured clinical interview that allows the formulation of a multiaxial psychodynamic diagnosis through the following axes: (axis I) experience of the disease and prerequisites for treatment, (axis II) interpersonal relationships, (axis III) conflict, (axis IV) structure, and (axis V) mental and psychosomatic disorders. In axes I, II, and IV, criteria that induce the coding of the interview as “0—absent, 1—mild/insignificant, 2—moderate, 3—high/significant, 4—very severe/very significant, and 9—not evaluable” are used. Axis II shows 32 patterns of dysfunctional relationships, themes, and resources presented by the patient and scored by judges (Task Force, 2016). Detailed description can be found in the Supplementary Table S1.

### Ethical procedures, data collection, and analysis

The study was approved by the ethics committee of the Federal University of Rio Grande do Sul (CAAE 68271917.7.0000.5347, No. 2.412.749) and authorized by the Specialized Center where data were collected. Participants signed the Free and Informed Consent Form. Data collection was carried out in a clinical care room with researcher and participant. The psychologist researcher has thirteen years of experience in psychodynamic psychotherapy and specific training to apply the OPD-2 clinical interview and had no previous knowledge of participants. The questionnaire was answered and later interviews took place, which were audio recorded, totaling 5 h and 20 min. Interviews were operated by two independent judges with agreement on all axes above 0.75, proving to be substantial.

Descriptive analysis was performed to characterize the sample and identify similarities and peculiarities in reports. According to Bardin (2008), analysis categories were created a posteriori. Results were compared with the existing literature relevant to the topic, scientific productions on PTSD and CPTSD, international psychodynamic studies and local investigations, since the occurrence of traumatic events has social attributes that should be considered.

## Results

### Sociodemographic and clinical characterization of participants

Participants were ten women. All were white and with mean age of 40 years (SD = 13.49). Psychiatric diagnoses were defined based on experienced symptoms, where participants 1, 2, 3, 4, and 5 met diagnostic criteria for PTSD (American Psychiatric Association, 2014) and participants 6, 7, 8, 9, and 10 were diagnosed with CPTSD (World Health Organization, 2018). Detailed data are found in Table 1.

**Table 1:** Sociodemographic and clinical data

Participants	Age	Schooling	Income	Psychoactive substances	Relationship
Participant 1	19	Incomplete high school	Between 1 and 2 minimum wages^a^	Do not use	Single
Participant 2	41	Incomplete primary education	Less than 1 minimum wage	Tabaco	Married
Participant 3	47	Incomplete primary education	Less than 1 minimum wage	Do not use	Married
Participant 4	51	Incomplete primary education	No income	Alcohol	Girlfriend
Participant 5	21	Complete primary education	No income	Do not use	Single
Participant 6	27	Complete primary education	Between 1 and 2 minimum wages	Do not use	Single
Participant 7	60	Complete high school	No income	Do not use	Widow
Participant 8	48	Technical education	Between 1 and 2 minimum wages	Do not use	Married
Participant 9	45	Incomplete primary education	No income	Do not use	Married
Participant 10	43	Incomplete primary education	No income	Do not use	Married

^a^Minimum monetary payment, defined by law, that a worker must receive for services rendered

### Thematic categories

Based on the analysis of interviews, categories reason for seeking care, symptoms, and desire for treatment, Traumatic developmental experiences and characteristics of psychic functioning were formed (detailed description can be found in Supplementary Table S2). It was observed that some aspects analyzed end up by being included in more than one category, as there is no way to isolate the dynamics into independent thematic modalities.

#### Reason for seeking care, symptoms and desire for treatment

This category records reasons for seeking specialized mental health care, symptoms developed after the index traumatic event and type of expected treatment. It is noteworthy that the index traumatic event is indicated as a triggering symptom of trauma.

The reasons for the initial search for care are similar for most participants, physical and emotional symptoms triggered after an index traumatic event: sexual violence, urban violence, accident with family member, and fire. Participant 10 points out that she sought care due to an “accumulation” of traumatic experiences in her development, emotional, sexual and domestic violence.

However, participants 2 and 5, despite bringing reports of traumatic experiences in their lives, are unable to associate them with their symptoms, seizures, and loss of leg movement, respectively. Such symptoms were understood in psychiatric diagnoses as somatic manifestations and not as neurological conditions. Participant 5 reported: “No, I do not know, I have never had an accident”.

It is noteworthy that, in most cases, the experience of trauma was not associated with the perception of symptoms, requiring referrals to professionals from other clinical specialties or the intervention of family members. Only participants 1 and 3, with PTSD, sought help on their own, identifying problems in their functioning.

In all cases, there is predominance of PTSD-related symptoms, flashbacks, hypervigilance, persistent feelings of threat, causing sleep difficulties, and distressing dreams. Fear, anxiety, anguish, and even panic seem to arise for no apparent reason or when related to activities and memories of the trauma. Somatic symptoms such as headache, abdominal pain and fatigue are reported. In addition, participants with CPTSD pointed to negative self-images, difficulties in interpersonal relationships, and persistent affect regulation problems. In addition, sense of loss of value and feelings of shame in the face of traumatic experiences are also observed.

In this sense, despite the severity of traumatic events and triggered symptoms, participants with PTSD were able to maintain, despite some difficulties, activities in the personal, social and occupational spheres. However, participants with CPTSD, who already showed global functioning with moderate to severe difficulties before the index traumatic event, had their symptoms intensified, abandoning most of their activities.

It is pointed out that suicidal ideation was present in half of participants, regardless of diagnosis. Even more serious, participants 2 and 10 had made suicide attempts, and according to participant 2: “I was sick, I took so many medications and cut myself in my arms”.

Her wish regarding treatment is the reduction of symptoms through medication. Participants 7 and 10 perceive benefits in being able to speak and think through psychotherapeutic treatment. “It is hard to speak out, but I leave here lighter” (participant 7).

#### Traumatic developmental experiences

This category brings traumatic experiences lived by participants throughout their lives, in their development. Experiences related to family and social contact were included.

In the life history of all participants, there are traumatic situations during childhood such as intra-family violence, fights and arguments between parents, as well as verbal and physical aggression. Such behaviors triggered feelings of rejection and affective withdrawal from their caregivers.

In addition, six participants reported episodes of childhood sexual violence caused by a family member. Participants 2 and 6 by their fathers and the others by brothers-in-law, cousins, or uncles. All of them reported that their caregivers, especially mothers, knew about the violence suffered; however, as they had a link with the aggressor, they neglected the violent episode and did not file any legal complaints. Consequently, some participants felt unassisted and were even abandoned by caregivers, starting to live with people outside the family nucleus. Participant 9 reported: “When I was abused, my mother sent me to a friend's house. I had to clean and cook. It was difficult, I did not understand why she gave me away”.

Among participants, only participants 1 and 3 reported to have had caregivers with stable affective behavior, capable of helping in their anxieties and to build an independent self. Participant 1 highlighted: “My father always supported me, he was always by my side, he gave me affection and explained things.

It was also evident the recurrence of traumatic events throughout their lives, sexual and urban violence and also intimate partner violence. In addition, they witnessed violence against other people, sexual violence against family members, violent death of close person and house fire, which were reported as traumas.

Faced with the violation of their rights, none of participants who suffered sexual violence or intimate partner violence made a judicial referral. In contrast, those who experienced urban violence registered a police report. Participant 10 reported: “I was very scared, but I went to the police station and told them what I had experienced”.

#### Characteristics of psychic functioning

The general characteristics of the levels of structural integration of personality, the main conflicts and their modes of organization, the defense mechanisms used and also the way in which interpersonal relationships are established, are described.

The personality structure refers to the self and its availability in the regulation of relationships with internal objects (autonomous self, internalization of good objects, self-regulation, self-reflection) or external-perception of the other and the realized, empathy (Task Force, 2016). In participants with PTSD and CPTSD, an important difference was observed in terms of structural personality functions. In most participants with CPTSD, the regulatory capacity dimension (self-regulation and regulation of the object relation) had low level and tendency to disintegration in relation to the object relation regulation. Thus, for the Task Force (2016), aggressive impulses coming from the Id cannot be integrated or blocked in the total aspect of the behavior, given the absence of normative instances, and the destructive hatred is justified by the actions of the others, without differentiation between their experiences, feelings, and interests. Participant 9 revealed: “The swing bothers me. Then she was there (my daughter) swinging, then I started screaming, slapped her in the face and took her home”.

However, in participants with PTSD, object perception ranged from moderate to low, where differentiation between their impulses and interests and those of others is not absent but reduced, with limited capacity to anticipate others’ reactions. It could be perceived in the speech of participant 5: “For God’s sake, they tell me, why are you so rude, she told me, but I do not understand why. In fact, I would rather be alone.” Furthermore, self-regulation did not vary in both diagnoses, being expressed at moderate to low level, in which instinctual desires are poorly tolerated, with low possibility of postponing or displacing satisfaction.

The other structural, cognitive (self-perception and object perception), emotional (internal communication and communication with the external world), and attachment capacity (internal objects and external objects) functions varied from moderate to low levels in all participants with reduced capacities and functions, marking the permanent tension in relation to their interior with difficulties to experience their own affections and fear of losing the object (Task Force, 2016). Thus, in general, the total personality structure of participants with PTSD and CPTSD is at moderate to low level; however, there is tendency of disintegration in the regulation of the object relation in participants diagnosed with CPTSD.

Likewise, the characteristics referring to psychic conflicts also differed among participants. In psychoanalysis, psychic conflict is manifested when there are opposing internal demands, which can be manifest (desire and moral demand) or latent. Such conflicts can manifest in the formation of symptoms and behavioral disorders (Laplanche & Pontalis, 1991). Participants with PTSD exhibited as main conflict the need to be taken care of versus Self-sufficiency, where they need the certainty of attention and care for the other. Participant 6 reported: “It is difficult, what I do is never good, they always leave me aside”. In participants with CPTSD; however, Individuation versus Dependence was identified as the major conflict, with more primitive developmental issues related to existential need. Participant 2 reported: “I need him, I cannot do it alone, I do not know what to do, I am nobody without him”.

In view of their psychic structures and conflicts, manifestations of protection of the self seek to control the anguish arising from internal aggressions (of instinctual order) and external sources of contempt, such as trauma, which can generate excitement (Laplanche & Pontalis, 1991). Thus, the most used defense mechanisms are linked to mental inhibition such as Affective Isolation and Dissociation (Clarkin et al., 2013; Task Force, 2016). Participant 2 reported “Sometimes I even lose my memory, I am in that place and I stay like that for half an hour, and I do not know where I am”. There was also the occurrence of denial of unpleasant facts through rationalization: “My mother was pregnant when my father died. So she forced herself to give it to me. I was one of the oldest” (participant 5) and defense actions that deal with internal or external stressors by action or withdrawal, such as Acting out: “I had so much anger, anger inside me. I once threw myself in front of a car” (participant 6). In addition, Somatization: “It was good, but when I got up in the morning I did not feel anything in my legs anymore (...). Both feet were bent (participant 2).

This set of characteristics of the participants’ psychic functioning acts on the way in which interpersonal relationships developed. Dysfunctional relationships with little proximity were evidenced, where participants do not feel understood, the others are felt as imposing themselves in a rude way, censoring or neglecting. In addition, sudden mood changes and avoidance of physical proximity increase interpersonal difficulties. Participant 10 reported: “It is getting more difficult every day, for me it is a sacrifice to be with someone. Nobody helps me”. The loss of interest also extends to daily activities like study, household chores, television programs, and they stop doing them, starting not feeling or experiencing positive emotions. Participant 7: “I do not have any joy, I do not want anything, I do not take care of my pets anymore”.

## Discussion

The study was carried out with ten women who sought a public service specialized in trauma assessment and treatment. At initial consultation, diagnosis of PTSD and CPTSD was evidenced. The sociodemographic findings confirm data provided by DSM-5 that describe patients with traumatic disorders as having tendency towards lower schooling, lower socioeconomic status, and less prevalent behaviors related to the use of psychoactive substances (American Psychiatric Association, 2014).

Through the OPD-2 clinical interview, experiences with care ambivalence, emotional, physical, and sexual violence were identified in their life histories, generating feelings of anger, fear, and abandonment. In such a way, the formation of insecure attachment seems to hinder the self-object differentiation, the regulation of the instinctual discharge and, consequently, the capacity for reflection and reorganization in the face of stress (Cryan & Quiroga, 2016). In view of this, the psychic structures of participants when experiencing the index traumatic event cannot manage the intensity of energy that is released, producing symptoms and disorders. Despite causing dysfunctions in different areas, such symptoms maintain some control over the mobilized anguish. Regarding specific sexual violence in childhood, Turner et al. (2017) point out that trauma becomes an uninterrupted horror when perpetrators have close relationship with victims, when they are not removed from conviviality. Blaming victims, denying, or minimizing the event and the effects of violence still tend to cause a poly-victimization potential.

Differently, participants 1 and 3 mentioned parents with the ability to welcome and translate childhood anxieties, the development of secure attachment and the internalization of stable representations, helped in the structuring of a representational model of the self with greater integration (Eizirik et al., 2015; Task Force, 2016). In this way, they seem to be able to better regulate the stress produced by the index traumatic event, enabling them to actively face their difficulties. Winnicott (1993) understands that the primary caregivers are a reference for the establishment of protection from trauma. In addition, these participants were the only ones to seek care, perceiving their emotional and behavioral difficulties after experiencing the trauma.

In general, psychic disorganization was identified in the vast majority of participants, with personality structures at moderate to low level of integration, indicating impaired perception of themselves and others, where instinctual impulses end up discharged in a poorly elaborated way. Thus, the intense fear of loss or separation from objects prevails, causing them to practice unconscious efforts of power and submission, with predominance of rigid and automatic assumptions (Sharp et al., 2016). In stressful situations, these representations become even more fragile, hindering the ability to self-regulate and making the presence of the other in a real way necessary (Task Force, 2016).

It is noteworthy that participants with CPTSD were identified as having severe commitment, with tendency to disintegration related to the object relation regulation. This marks an undifferentiated self, in which behaviors have no sense of authorship or normative instances, that is, they simply occur. Aggressive impulses are not recognized, but experienced as a justified reaction to the behavior of the other. Thus, the other, his/her interests and desires are not considered (Task Force, 2016).

Some authors already identified that emotional regulation difficulties were related to the development of PTSD symptoms (Bardeen et al., 2013) and that personality traits could be predictive for the development of the disease (DiGangi et al., 2013). Baie et al. (2020), in an investigation specifically on the personality structure of patients with PTSD, reported that the lower the structural level of integration of the subject’s personality, the more pronounced the symptoms of the disease. His study, however, did not evaluate CPTSD patients, in which according to ICD 11 (2018), symptoms are more severe and generalized and focused on the organization of the self-differentiation and regulation, as pointed out in this study.

Corroborating the structural level findings, there were differences regarding the main psychic conflicts between PTSD and CPTSD participants. Women with PTSD presented the conflict of need to be taken care of versus self-sufficiency, in which the image of the other is present in a fragile way, with fear of its loss. Thus, they constantly seek to have the attention of the other and his/her care, obtaining benefits and understanding them as security. Physical symptoms or diseases offer a legitimation of their dependence (Task Force, 2016). However, participants with CPTSD presented the conflict of individuation versus dependence, in which the self is confused with the other, being undifferentiated. In such a way, the annihilation anguish is constant, with a feeling of not existing on its own. Thus, it was observed that participants with CPTSD present greater impairments in their levels of functioning, greater severity of symptoms and worse quality of life.

Regarding the personality structure and psychic conflicts presented, all participants, regardless of diagnosis, used low qualitative and effectiveness variations in relation to the ego defenses with predominant immature development factor involving mental inhibition and denial of reality. For Gabbard (2016), this inflexible defense pattern would imply worse capacity to adapt to stressful events. Thus, according to other studies with PTSD patients, through affective isolation, they seek to separate the image of the traumatic event and the distressing affects produced in an attempt to avoid unbearable stress (Paulo & Pires, 2013; Teche et al., 2017). Or, through the defense mechanisms of dissociation, they try to separate potentially threatening thoughts and desires from consciousness, reducing anguish and the feeling of helplessness (Gabbard, 2016; Task Force, 2016).

Unlike MØller et al. (2021), who identified associations between dissociative phenomena only in patients with CPTSD, this study identified its use in all participants. Also, the use of Somatization and Rationalization has been registered as a way of converting emotional pain into physical symptoms, changing the focus of concerns (Laplanche & Pontalis, 1991) such as headaches, chest, and abdomen pains, trying to justify their dependence, impulsive behaviors faced with the attitudes of others, sudden mood changes, sometimes hypervigilant, irritable or aggressive, sometimes disinterested, alien, or unable to feel satisfaction.

Faced with this psychic organization with primitive constitution issues, participants demonstrate in their current interpersonal relationships a repetitive model of approximation, fear of fusion, distancing, and constant rupture. In view of this, tendency of dyadic relational patterns of dependence and need is observed, where the other is a significant object of support and regulation, remaining in relationships for a long period even if they are submitted or neglected. However, they seek to identify a tolerable distance, given the fear of merging with the object, their own destruction or the destruction of the other. However, in the anguish intensification, they end up by breaking relationships. This distancing, however, is also intolerable, given the reactivation of feelings of helplessness and instinctual anguish from the first moments of childhood (Task Force, 2016).

With inadequate coping strategies, the use of acting out manifests itself with self-destructive behaviors, such as suicide attempts. It is known that people with PTSD are more likely to develop a suicidal plan than people without the disease and that women are at higher risk (4.3%) when compared to men (2.3%) (O’Neill et al., 2014). In this sense, the severity of the disorder, its symptoms, and subjective suffering were evident where half of participants alluded to suicidal ideation and two tried to commit suicide on more than one occasion.

The reasons for seeking treatment or referrals were complaints related to symptoms and the desire to reduce them through medication, with low availability to talk about themselves and little capacity to relate them to traumatic situations. However, with the psychodynamic assessment through the OPD-2 clinical interview, it was possible to understand traumatic events in their life histories, constitution of psychological and social resources, in addition to the identification of the different modes of functioning in each of the traumatic disorders.

The possibility of building a safe space to tell their histories, their suffering and to be heard are the basis for the establishment of trust and therapeutic alliance, fundamental for treatment. However, one should be aware of movements of premature interruptions and abandonment, given characteristics related to feelings of danger and instabilities in relationships (Outcalt et al., 2016). Regarding patients with greater psychic disorganization, as observed in these participants, it is necessary to strengthen the ego functions so that they can deal with reality, where the psychotherapist places himself as a real person and not just as a transference object (Zimerman, 1999). In addition to education about symptoms and the nature of PTSD, expectations, fears, and resistances must be clarified, with empathy and compassion (Martínez, 2019).

Especially for patients with CPTSD, the therapeutic focus should be on the structure, as difficulties are related to limitation in development, with severe frailties of the self. Thus, the therapeutic relationship must seek to build elements that are absent in the patient’s history, in addition to containing emotions. Communication should not be just verbal, but through the therapist’s behaviors, transmitting security and truth. Representing a new primary attachment figure, the patient should be directed towards autonomy, offering new connections capable of changing poorly adapted relational patterns, starting to relate to good objects and using resources from external relationships. The hypotheses must be offered by looking at their later formulations (Task Force, 2016).

## Limitations and future researches

Despite the contribution of this study on traumatic disorders in southern Brazil, it has some limitations. Since its cross-sectional design demonstrates the current situation of participants, it is not possible to show how symptoms and treatments evolved in the different participants and different disorders. Interviews were analyzed by audio transcription by two judges in order to minimize the subjective influence of the researcher. Expressiveness was kept in report, such as crying and feelings of anger, among others. Data were based on the COREQ guidelines (Tong et al., 2007) for greater consistency among methodologies. Regarding the sample size, the criterion of data saturation was used, seeking to be consistent with reality; however, they should not be seen as conclusive. Future researches on the different disorders may expand data in the psychodynamic perspective related to trauma. In addition, longitudinal studies could obtain information about post-traumatic growth and trauma perpetuation throughout life.

## Conclusions

In general, the results indicate that the use of psychodynamic assessment in trauma patients, based on OPD-2, can be essential to understand differences and peculiarities in their psychic organizations and help health professionals in the direction of treatment. It was found that participants with CPTSD presented in their psychic structures tendency to disintegration related to the regulation of the object relation and the psychic conflict was individuation versus dependence, which demonstrates failures in the positive representation of objects and in the capacity to differentiate the object in face of their existential need, in addition to the direct discharge of impulses without sense of authorship. Participants with PTSD had moderate to low level of integration of their structures and the conflict of need to be cared for versus self-sufficiency, with reduced perception of the internalized object, fragile self-representations, and intense need for attention and care (Task Force, 2016).

It was possible to identify a cycle of adverse events in the participants’ lives, causing psychic organization with certain disintegration, difficulties in emotional regulation and, consequently, in reflection. Later traumas further disorganize the psychic structure, intensifying anguish, search for control in relation to the other, isolation, and somatic symptoms.

Such assimilation makes it possible to consider risk factors for the development of disorders, patterns of transgenerational violence, urban violence, and psychic organization. In addition, diversity of symptoms and the scope of affected areas in the life of the individual are observed, which should guide actions for the non-consolidation of the disease, involving the early identification of traumas and new ways of offering support to patients, creating a space for talking, listening, and thinking, and identifying psychological and social resources and obstacles to treatment. The training of public health services for early identification and action in cases of traumatic situations and Brief Analytical Orientation Psychotherapy (Eizirik et al., 2015) or cognitive-behavioral interventions (Cloitre et al., 2002), works focused on interpersonal skills and compassion (Karatzias et al., 2019), in addition to psychotropic drugs when necessary, have shown significant results. Aspects to prevent violence must be developed, such as actions to guide parents and caregivers and the deconstruction of cultural and social aspects related to the naturalization of aggression, especially in childhood. Likewise, understanding work as a promoter of quality of life, organizations must have worker health policies aimed at prevention and recovery in cases of exposure to or witnessing stressful events, thus avoiding absenteeism, leaves, or work abandonment. Specific works with aggressors must be performed, reducing the possibilities of reproducing violence.

## Supplementary Information

**Additional file 1: Table S1.** Axes definition, classification, alignment and reference description according to OPD-2.

**Additional file 2: Table S2.** Comprehensive description of categories.

## Data Availability

The data analysed in the study are detailed in the Complementary Tables. Table S1 briefly describes how the psychodynamic diagnosis of the interviews was performed. Table S2 provides a detailed description of the Categories based on data from participant interviews. Other data and materials may be made available upon request to the corresponding author upon reasonable request.

## Abbreviations

PTSD: Post-traumatic stress disorder
CPTSD: Complex post-traumatic stress disorder
OPD-2: Operationalized Psychodynamic Diagnosis-2
DSM-5: Diagnostic and Statistical Manual of Mental Disorders
ICD 11: International Classification of Diseases for Mortality and Morbidity Statistics
