# Supplementary material for: Understanding the psychodynamic functioning of patients with PTSD and CPTSD: qualitative analysis from the OPD 2 interview
Source: Psicol Reflex Crit. 2022 Apr 18;35:9. doi: 10.1186/s41155-022-00211-5 (PMC9016102; doi:10.1186/s41155-022-00211-5)
Supplement: Supplementary file 1 — Additional file 1: Table S1. Axes definition, classification, alignment and reference description according to OPD-2. [file 41155_2022_211_MOESM1_ESM.docx]

**Table S1**

Axes definition, classification, alignment and reference description according to OPD-2

| **Axes** | **Classification** | **Encoding** | **Reference description** |
| --- | --- | --- | --- |
| I-Disease development and prerequisites for treatment | - current disease severity  - duration  - form of presentation and conceptualization by the patient  - resources and resistance to change | 0- nothing/rarely present  1- light/insignificant  2- moderate  3- high/significant    4- very serious/very significant  9- non-evaluable | Levels of symptoms/desire for treatment:  - physical and mental symptoms restricted  - no desire for physical and/or mental treatment  - mild physical and mental symptoms  - low desire for physical and/or mental treatment  - symptoms and relevant limitations in the physical and/or mental domain  - desire for physical and/or mental treatment  - severe symptoms and significant limitations in the physical and/or mental domain  - significant desire for physical and/or mental treatment  - extensive physical and/or mental symptoms  - very high desire for physical and/or mental treatment  - there is no diagnostic safety for evaluation |
| Axis II- Interpersonal relationships | Dysfunctional relationship patterns, themes and resources are established. In perspective A, the experience by the patient and in perspective B, the perception of others | 32 items of interpersonal relationships | Relationship diagnosis scheme  Perspective A: the patient's experience  I- how the patient often experiences himself (response experienced as defensive)  IV-how the patient experiences others frequently (experienced attack/disappointment)  Perspective B, the perception of others  II- others, including the interviewer experience the patient frequently (offer of the relationship)  III- others, including the interviewer, experience themselves in relation to the patient. |
| Axis III - Conflict | - Fundamental motivation that emerges throughout life as a continuum reflecting individual patterns of experience and behavior.  repetitive dysfunctional disorders are scored according to intensity. The main conflict and the secondary conflict are the most significant.  - The ways in which conflicts, active or passive, are processed.  1-Individuation versus Dependence  2- Submission versus Control  3- Need to be careful versus self-sufficiency  4- Self-esteem Conflict  5- Conflict of guilt  6- Oedipal conflict  7- Identity conflict | 0-nothing/rarely present  1-light/insignificant  2-moderate  3- high/significant  4- very serious/very significant  9- non-evaluable  0-nothing/rarely present  1-light/insignificant  2-moderate  3- high/significant  4- very serious/very significant  9- non-evaluable  0-nothing/rarely present  1-light/insignificant  2-moderate  3- high/significant  4- very serious/very significant  9- non-evaluable  0-nothing/rarely present  1-light/insignificant  2-moderate  3- high/significant  4- very serious/very significant  9- non-evaluable  0-nothing/rarely present  1-light/insignificant  2-moderate  3- high/significant  4- very serious/very significant  9- non-evaluable  0-nothing/rarely present  1-light/insignificant  2-moderate  3- high/significant  4- very serious/very significant  9- non-evaluable  0-nothing/rarely present  1-light/insignificant  2-moderate  3- high/significant  4- very serious/very significant  9- non-evaluable | - Passive mode: effort to establish close and secure relationships. Responsibilities avoided. Personal desires subordinated by the perception of impotence and weakness.  - Active mode: efforts to build exaggerated relationships and existential independence. Constant struggle for autonomy and independence. Conviction of not needing anyone.  - Passive mode: passive-aggressive submission. Main affect is anger with feelings of subjugation.  - Active mode: Seeking control over others and situations, with devaluation and insults to banish the fear of being controlled. Aggressive affection.  - Passive mode: Need for security and care. Fear of rejection and of losing the other. Dependent, attached and demanding. Envy is frequent.  - Active mode: Concern for the other to cover latent depressive feelings. It seeks to surround itself with people to stabilize the self.  - Passive mode: Critical immersion in self-esteem with feelings of worthlessness, unimportance and shame. Idealized admiration for the other.  - Active mode: vigorous self-confidence in others as an attempt to deal with real or feared self-esteem. Irritability and anger when your narcissistic image is questioned.  - Passive mode: accepts guilt in an exaggerated and constant way with a submissive and selfless attitude. Fear of loss, punishment and sadness can emerge.  Active mode: Feelings of guilt are denied, repressed and passed on to others. This results in anger towards others.  - Passive mode: They do not feel admired, recognized, or sexually attractive because of their physical or character attributes. Feelings of inferiority and submission where they repress their sexuality.  - Active mode: Erotically inappropriate behavior. They seek attention for themselves to shine and seduce.  - Passive mode: chronic feeling of lack of identity. Avoidance of situations that confront the subject with insecurity about their identity.  - Active mode: Hides your insecurities in your own identity, avoiding dissonance. It seeks to build a family saga with “borrowed” identities. |
| Axis IV - Structure  1- Cognitive abilities: Self-perception and object perception | The availability of mental functions in the regulation of the self and its relationships with internal and external objects are evaluated.  1a- Self-perception | 1-High  1.5- High to moderate/Medium  2- Moderate/Average  2.5- Moderate to low  3- Low  3,5- Low disintegration  4- Disintegrated  9- Not available | - Constant image over time. Differentiates affections. Ability to deal with intrapsychic conflicts. Regulatory functions available even in voltage generating cases. Fear of the loss of the abject's care and attention.  - More destructive psychic conflicts. Tendency to self-devaluation. Self-image and identity difficulties.  - Poorly developed psychic space and differentiation from limited structures. Interpersonal and not intrapsychic conflicts. Lack of ability to think about yourself. Fuzzy identity. Negative Affects. Impulsive explosions.  - Lack of coherence of the self, with images between self and object being confused. Defensive patterns cover up post-psychotic, post-traumatic, or perverse organization. Responsibility for impulsive actions are not experienced.  - there is no diagnostic safety for evaluation |
|  | 1b- Perception of the object | 1-High  1,5- High to moderate/Medium  2- Moderate/Average  2.5- Moderate to low  3- Low  3,5- Low disintegration  4- Disintegrated  9- Not available | - Affects, impulses and thoughts are perceived differently as to their attribution to the self or to objects. Good enough internal objects.  - Images of objects are limited. Reduced empathy ability. Predominance of dyadic relationships. Major fear of object loss  - Persecuting and punishing internal objects. Fear of annihilation of the self through loss of the good object or through the bad object.  Low to crumbled.  - Empathic object perception is almost impossible. Main fear of symbiotic fusion between self and object.  - there is no diagnostic safety for evaluation. |
| 2- Capacity for regulation | 2.1- Self regulation | 1-High  1.5- High to moderate/Medium  2- Moderate/Average  2.5- Moderate to low  3- Low  3,5- Low disintegration  4- Disintegrated  9- Not available | - Built-in instinctual drives that can be controlled. Ambivalences are tolerated and compromises are sought. Self-esteem can be maintained or recovered.  - Judgment of value is reduced. Affections and desires on a conscious level and poorly tolerated. Low emotional flexibility. Difficulty in postponing or displacing satisfaction by impulse is reduced. Self-esteem is easily damaged.  - Low value judgment and low possibility of postponing or displacing impulse satisfaction. Self-dystonic behavior and rejected by the environment as inappropriately hostile. Regulation is abrupt and ineffective. Fragile self-esteem. Guilt becomes self-destructive. Ideas of unrealistic magnitude.  - There is no sense of authorship in their actions. Destructive hate explained by the actions of the other. Sexual drives based on partial drives. Self-criticism moves between grandiosity and degraded self-esteem. Inability to follow a goal.  - there is no diagnostic safety for evaluation |
|  | 2.2- Regulation of the relationship with the object | 1-High  1.5- High to moderate/Medium  2- Moderate/Average  2.5- Moderate to low  3- Low  3,5- Low disintegration  4- Disintegrated  9- Not available | - Relationship with another is not negatively affected by a defensive personal attitude or by your impulses. Ability to anticipate the behavior of others.  -nPersonal and other interests are not clearly perceived. Ability to anticipate reactions from others is limited. He feels exploited, often getting into trouble.  - Defensively interpersonal relationships. Cannot process needs and fears based on conflict, letting them spill over into the relationship. Interests of others are not taken into account, not regulating their behavior.  - Low possibility of identifying delineated objects and considering their interests or anticipating their behavior. |
| 3- Emotional capacity: internal communication and communication with the external world. | 3.1- Internal communication | 1-High  1,5- High to moderate/Medium  2- Moderate/Average  2,5- Moderate to low  3- Low  3,5- Low disintegration  4- Disintegrated  9- Not available | - Produce and experience their affections. Emotional and communicative contact in your relationships. Neurotic conflicts with feelings of fear, shame and guilt can delay communication but not suspend it.  - Ability to feel and generate affections and fantasies is limited.  - Difficulty recognizing friendly and tender feelings, but also sadness and feelings of guilt. Impulsiveness, described as despair, panic and anger. Body experience can be fragmented.  - Intense, disorganized and rigid affections that he cannot express. Fragmented fantasies, memories and affections. Alienated and strange bodily self. |
|  | 3.2- Communication with others | 1-High  1,5- High to moderate/Medium  2- Moderate/Average  2.5- Moderate to low  3- Low  3,5- Low disintegration  4- Disintegrated  9- Not available | - Empathy.  -Limited communication, rigid behavior and affect sensitive to emotional damage. Limited empathy.  - Unable to experience the other's inner world. Difficulty in distancing, often manipulating and going beyond limits.  Low empathy with using the other for individual purposes.  - There is no communicative exchange, the needs of others are ignored  - there is no diagnostic safety for evaluation |
| 4- Ability to cling: internal and external objects | 4.1- Ability to cling to internal objects | 1-High  1,5- High to moderate/Medium  2- Moderate/Average  2,5- Moderate to low  3- Low  3,5- Low disintegration  4- Disintegrated  9- Not available | - Positive object representations, with the ability to maintain affection and benefit, calming, comforting and protecting oneself.  Variable and triangular links.  - Variable internal images with the possibility of getting lost in conflict situations. Object dependency.  - Disturbed, threatening and persecutory internal images. Socially responsible attitudes towards internalized objects are absent. I fear that the self will be annihilated by the loss of the good object or the bad object.  - Undefined objects. Impulsiveness. Relationship with symbiotic fusion of self and object representations, with consequent loss of identity  - there is no diagnostic safety for evaluation |
|  | 4.2- Ability to attach to external objects | 1-High  1,5- High to moderate/Medium  2- Moderate/Average  2,5- Moderate to low  3- Low  3,5- Low disintegration  4- Disintegrated  9- Not available | - Capacity for external links with adequate approximation and distance. Prosocial affects.  - Limited emotional bond. Goodbyes are avoided which is covered up by excessive attachment.  - Fickle attachment. Excessive feelings of love, hate, or great distance.  - Cannot establish long-lasting bonds, creating feelings of strangeness and ignorance in relation to the interests of others.  - there is no diagnostic safety for evaluation |
| 5- Total structure | The total structure level is evaluated based on the general characteristics.  The most prevalent defenses at each structural level are observed. | 1-High  1,5- High to moderate/Medium  2- Moderate/Average  2,5- Moderate to low  3- Low  3,5- Low disintegration  4- Disintegrated  9- Not available | - Typical defenses: Repression, rationalization, displacement.  - Typical Defenses: Denial, Reactive Formation, Isolation, and Projection.  -bTypical defenses: Cleavage, pathological projective identification and idealization or devaluation of objects.  - Distortion of reality.  - there is no diagnostic safety for evaluation. |
| Eivo V- Mental and psychosomatic disorders. | Diagnosis according to DSM-V or ICD-10 |  |  |

*Note:* Description according to OPD-2 Operational Psychodynamic Diagnosis (Task Force, 2016). Produced by the authors.

**Bibliographic Reference:**

Task Force. (2016). *Diagnóstico psicodinâmico operacionalizado: Manual de diagnostico e plano de tratamento (OPD-2 )* [Operationalized psychodynamic diagnosis: Diagnostic manual and treatment plan (OPD-2)]. São Paulo: Hogrefe
