# Supplementary material for: Understanding the psychodynamic functioning of patients with PTSD and CPTSD: qualitative analysis from the OPD 2 interview
Source: Psicol Reflex Crit. 2022 Apr 18;35:9. doi: 10.1186/s41155-022-00211-5 (PMC9016102; doi:10.1186/s41155-022-00211-5)
Supplement: Supplementary file 2 — Additional file 2: Table S2. Comprehensive description of categories. [file 41155_2022_211_MOESM2_ESM.docx]

**Table S2**

Comprehensive description of categories

|  | Trauma, Symptoms and Diagnosis | Traumatic Developmental | Experiences Psychodynamic Functioning Characteristics |
| --- | --- | --- | --- |
| Participant 1 | Seeking care: difficulties in functioning  Symptoms: reliving memories  hypervigilant  Anxiety  Fear  Improvement of symptoms by drug treatment | Caring and loving parents | Structure: self-perception and object perception - moderate to low  self-regulating - moderate and low level  regulation of the object relation - moderate to low  built-in object linking and object linking - moderate to low  Conflict: need to be cared for versus self-sufficiency  MD: Affective Isolation  Rationalization    Relational patterns: accuse and neglect  Distance/approach difficulties  loss of interest in everyday activities  mood change  Keeps working |
| Participant 2 | Seeking care: family intervention  Sadness and discouragement before the traumatic event index  Symptoms: reliving memories  hypervigilant  Somatic symptoms  Anxiety  Panic  Suicidal ideation  Suicide attempts  Improvement of symptoms: drug treatment | Verbal aggression by caregivers  Sexual violence in childhood (father)  Intimate partner violence  sexual violence by stranger | Structure: self-perception and object perception - moderate to low  self-regulating - moderate and level  regulation of the object relation - moderate to low  internal communication and external communication with objects - moderate to low  built-in object linking and object linking - moderate to low  Conflict: need to be cared for versus self-sufficiency  MD: Dissociation  Rationalization  Acting out  somatization  Relational patterns: accuse and neglect  Loss of interest in everyday activities  loss of positive emotions  Withdrawal from work |
| Participant 3 | Seeking care: difficulties in functioning;  Sadness and discouragement before the traumatic event index  Symptoms: reliving memories  Somatic symptoms  Improvement of symptoms: drug treatment | Caring and loving parents  Sexual violence in childhood (brother-in-law)  sexual violence against family members | Structure: self-perception and object perception - ranged from moderate to low  self-regulating - moderate and low level  regulation of object-ratio-moderate to low;  internal communication and external communication with objects - moderate to low  internal communication and external communication with objects - moderate to low  built-in object linking and object linking - moderate to low  Conflict: need to be cared for versus self-sufficiency  Relational patterns: accuse and neglect  MD: Affective Isolation  somatization  Distance/approach difficulties  Keeps working |
| Participant 4 | Seeking care: family intervention;  Sadness and discouragement before the traumatic event index;  Symptoms: reliving memories;  Hypervigilant;  sleeping difficulties and distressing dreams;  anxiety;  fear;  Suicidal ideation;  Symptom improvement: drug treatment | Parental quarrels and arguments  Sexual violence in childhood (brother-in-law)  Death of close person violently | Structure: self-perception and object perception - ranged from moderate to low  self-regulating - moderate to low  regulation of object-ratio-moderate to low;  internal communication and external communication with objects - moderate to low  built-in object linking and object linking - moderate to low  Conflict: need to be cared for versus self-sufficiency  MD: Affective Isolation  Acting out  somatization  Relational patterns: accuse and neglect  Distance/approach difficulties  Loss of interest in everyday activities  loss of positive emotions  Withdrawal from work |
| Participant 5 | Seeking care: medical referral;  Anxiety before the traumatic event index;  Symptoms: difficulty sleeping and distressing dreams;  Suicidal ideation;  Improvement of symptoms: drug treatment | Parental quarrels and arguments  verbal aggression  physical aggression  affective distancing  abandonment  Intimate partner violence  sexual violence by stranger | Structure: self-perception and object perception - moderate to low  self-regulating - moderate to low  regulation of the object relation - moderate to low  internal communication and external communication with objects - moderate to low  built-in object linking and object linking - moderate to low  Conflict: need to be cared for versus self-sufficiency  MD: Affective Isolation  Dissociation  Rationalization  Acting out  somatization  Relational patterns: accuse and neglect  Distance/approach difficulties  Withdrawal from work |
| Participant 6 | Seeking care: medical referral  Symptoms: reliving memories  Hypervigilant  Fear  Anxiety  Low affect regulation  Beliefs of self diminished and worthless  Improvement of symptoms: drug treatment | Sexual violence in childhood (father)  Death of close person violently | Structure: self-perception and object perception - moderate to low  self-regulating - moderate and low  regulation of the object relation - tendency to disintegration  internal communication and external communication with objects - moderate to low  built-in object linking and object linking - moderate to low  Conflict: individuation versus dependence  Relational patterns: accuse and neglect  Distance/approach difficulties  Avoidance of physical approach  loss of positive emotions  Withdrawal from work  MD: Affective Isolation  Rationalization |
| Participant 7 | Seeking care: medical referral  Sadness and discouragement before the traumatic event index  Symptoms: reliving memories  Hypervigilants  Fear  Anxiety  Beliefs of self diminished  Low affect regulation  Suicidal ideation  Improvement of symptoms: drug treatment | Parental quarrels and arguments  verbal aggression  Rejection  live with other family members  Intimate partner violence | Structure: self-perception and object perception - moderate to low  self-regulating - moderate to low  regulation of the object relation- tendency to disintegration;  internal communication and external communication with objects - moderate to low  built-in object linking and object linking - moderate to low  Conflict: individuation versus dependence  MD: Affective Isolation  Dissociation  Acting out    Relational patterns: accuse and neglect  Distance/approach difficulties  Withdrawal from work |
| Participant 8 | Seeking care: medical referral;  Symptoms: reliving memories;  Difficulties sleeping  Distressing dreams;  Fear;  Anxiety;  Fragility;  Guilt beliefs;  Low affective regulation;  Improvement of symptoms: drug treatment | Fights and arguments  Rejection | Structure: self-perception and object perception - ranged from moderate to low  self-regulating - moderate to low  regulation of the object relation - moderate to low  internal communication and external communication with objects - moderate to low  built-in object linking and object linking - moderate to low  Conflict: individuation versus dependence  Withdrawal from people and difficulties in feeling close  MD: Acting out  somatization  Relational patterns: accuse and neglect  Avoidance of physical approach  Loss of interest in everyday activities  Withdrawal from work |
| Participant 9 | Search for care: medical referral  Anxiety before the traumatic event index  Symptoms: reliving memories  hypervigilant  Fear; Anxiety  Belief of worthlessness  Low affective regulation  suicidal ideation  Improvement of symptoms: drug treatment | Parental quarrels and arguments  Sexual violence in childhood (cousin)  Sexual violence in childhood (uncle)  Living outside the family nucleus  sexual violence against family members  Death of close person violently | Structure: self-perception and object perception - ranged from moderate to low  self-regulating - moderate and low level  regulation of the object relation- tendency to disintegration;  internal communication and external communication with objects - moderate to low  built-in object linking and object linking - moderate to low  Conflict: individuation versus dependence  MD: Affective Isolation  Dissociation  Relational patterns: accuse and neglect  Distance/approach difficulties  Avoidance of physical approach  Loss of interest in everyday activities  loss of positive emotions  Withdrawal from work |
| Participant 10 | Search for care: “accumulation” of trauma;  Sadness and discouragement before the traumatic event index  Symptoms: reliving memories  Hypervigilant  Difficulty sleeping and distressing dreams  Anxiety  Anguish  Panic  Fear  Somatic symptoms  Belief of worthlessness, shame and failure  Low affective regulation  Suicidal ideation  Suicide attempts  Improvement of symptoms: psychotherapeutic treatment | Parental quarrels and arguments  Rejection  Sexual violence in childhood (uncle)  live with strangers  Intimate partner violence  sexual violence by stranger  Urban violence (robbery)  Death of close person violently  home fire | Structure: self-perception and object perception - ranged from moderate to low  self-regulating - moderate and low level  regulation of the object relation - tendency to disintegration  internal communication and external communication with objects - moderate to low  built-in object linking and object linking - moderate to low  Withdrawal from people and difficulties in feeling close  Conflict: individuation versus dependence  MD: Affective Isolation  Dissociation  Rationalization  Acting out  somatization  Relational patterns: accuse and neglect  Loss of interest in everyday activities  loss of positive emotions  mood change  Avoidance of physical approach  Withdrawal from work |

*Note:* produced by the authors.
